# Comparative Analysis of Conventional Natural Killer Cell Responses to Acute Infection with *Toxoplasma gondii* Strains of Different Virulence

**DOI:** 10.3389/fimmu.2016.00347

**Published:** 2016-09-23

**Authors:** Daria L. Ivanova, Rida Fatima, Jason P. Gigley

**Affiliations:** ^1^Department of Molecular Biology, University of Wyoming, Laramie, WY, USA

**Keywords:** *Toxoplasma gondii*, toxoplasmosis, natural killer cells, virulence, cytokines

## Abstract

Conventional natural killer (cNK) cells, members of group 1 innate lymphoid cells, are a diverse cell subpopulation based on surface receptor expression, maturation, and functional potential. cNK cells are critical for early immunity to *Toxoplasma gondii via* IFNγ production. Acute cNK cell responses to infection with different strains of *T. gondii* have not yet been characterized in detail. Here, we comprehensively performed this analysis with Type I virulent RH, Type II avirulent ME49, and fully attenuated Type I *cps1-1* strains. In response to these three parasite strains, murine cNK cells produce IFNγ and become cytotoxic and polyfunctional (IFNγ+CD107a+) at the site of infection. In contrast to virulent RH and avirulent ME49 *T. gondii* strains, attenuated *cps1-1* induced only local cNK cell responses. Infections with RH and ME49 parasites significantly decreased cNK cell frequency and numbers in spleen 5 days post infection compared with *cps1-1* parasites. cNK cell subsets expressing activating receptors Ly49H, Ly49D, and NKG2D and inhibitory receptors Ly49I and CD94/NKG2A were similar when compared between the strains and at 5 days post infection. cNK cells were not proliferating (Ki67−) 5 days post infection with any of the strains. cNK cell maturation as measured by CD27, CD11b, and KLRG1 was affected after infection with different parasite strains. RH and ME49 infection significantly reduced mature cNK cell frequency and increased immature cNK cell populations compared with *cps1-1* infection. Interestingly, KLRG1 was highly expressed on immature cNK cells after RH infection. After RH and ME49 infections, CD69+ cNK cells in spleen were present at higher frequency than after *cps1-1* infection, which may correlate with loss of the mature cNK cell population. Cytokine multiplex analysis indicated cNK cell responses correlated with peritoneal exudate cell, spleen, and serum proinflammatory cytokine levels, including IL-12. qPCR analysis of parasite-specific B1 gene revealed that parasite burdens may affect cNK cell responses. This study demonstrates infection with RH and ME49 parasites impacts cNK cell maturation during acute *T. gondii* infection. Different cNK cell responses could impact early immunity and susceptibility to these strains.

## Introduction

*Toxoplasma gondii* is a highly prevalent food-borne obligate intracellular parasitic protozoan present in 30% of humans, which is a significant health concern as an opportunistic infection in immunocompromised people (1). Health outcomes after infection depend on many factors, including parasite genotype. In North America and Europe, *T. gondii* strains are represented by frequently found type II, III, 12 strains of a low virulence (LD50s of ~10^3^, 10^5^, 10^3^ parasites, respectively) and less common but highly virulent type I strain (100% lethal dose [LD100], 1 parasite) (2). Parasite virulence can affect how well the immune system responds, leading to differences in infection pathology (3). Thus, understanding how different parasite strains impact immune response is critical to improve therapies and vaccines to combat this infection.

Control of acute and chronic *T. gondii* infection is mediated by Th1 cell-mediated immunity (4). Conventional natural killer (cNK) cells are critical for innate immunity to *T. gondii* by producing IFNγ (5, 6). cNK cell IFNγ production is dependent upon IL-12 (6). cNK cells have also been shown to have an important helper role in stimulating adaptive immunity to *T. gondii*. In CD4 T cell-deficient mice cNK cell IFNγ can help induce CD8 T cell immunity to *T. gondii* (7). IFNγ produced by cNK cells also promotes development of inflammatory dendritic cells, which, in turn, activates T cell responses (8). cNK cells also show cytotoxic activity in a response to *T. gondii* parasites and their subcellular components (9–11). However, the importance of cNK cell cytotoxicity during *T. gondii* infection is still not known (12).

Conventional natural killer cells are innate immune cells important for early control of cancer and infectious pathogens. They are members of the newly named group 1 ILC population and develop in the bone marrow from the common lymphoid progenitor (13). cNK cells provide protection by producing pro-inflammatory cytokine IFNγ and cytolytic activity. The activation of cNK is dependent upon the signals generated by activating and inhibitory receptors (14, 15). Activating receptors include those that recognize specific ligands expressed on the surface of target cells, Ly49H, Ly49D, and NKG2D, as well as cytokine receptors for IL-12 and Type I IFNs. Inhibitory receptors recognize classical and non-classical MHC class I molecules that are also expressed on the surface of target cells and include Ly49I and NKG2A. *Via* these receptors, cNK cells are turned on to provide immunity in many disease situations.

Engagement of receptors by specific ligands impacts the fate and composition of responding cNK cells (16). For example, Ly49H activating receptor expressing cNK cells specifically recognize m157 proteins on MCMV-infected cells and develop memory response to subsequent MCMV infections (17). In human studies, cNK cells that express NKG2C/CD94 heterodimer expand in a response to HCMV (18) and other viruses, such as HIV (19–21), Hantavirus (22), and Chikungunya virus (23). Whether a dominant cNK cell population is associated with *T. gondii* infection is not clear. Additionally it is not known whether cNK cell population composition is affected by the infection with different *T. gondii* strains.

The functional potential of cNK cells can be dependent on cNK cell maturation (24). cNK cells progress through a 4-stage developmental program defined by the expression of CD27 and CD11b (25). Highly mature cNK cells (CD27−CD11b+) acquire complete functional potential, are able to migrate, and lose their proliferative potential (24). cNK cell maturation can be affected by the signals received during infection (26). The maturation profile of cNK cells during *T. gondii* infection is not known and is important to define to gain a better understanding on how infection may impact cNK cell development.

The question of how different *T. gondii* strains can affect cNK cell biology has not been thoroughly addressed. In this study, we pursue this question and find that infection with virulent RH and avirulent ME49 parasites negatively impacts specific aspects of cNK cell responses to infection. Regardless of parasite strain and the parasite load, cNK cells increase in the site of infection. This increase corresponds with increase in cNK activation and function. The attenuated Type I strain *cps1-1* stimulates only a localized cNK cell response compared with parental RH strain and Type II strain ME49. RH and ME49 infections significantly reduce splenic cNK cell numbers. Despite differences in the magnitude of cNK cell responses, cNK cells appear to respond in a global manner with no distinct subpopulation bearing specific receptors. Parasite virulence appears to have an impact on cNK cell maturation where highly virulent RH strain results in a significant loss in mature (CD27−CD11b+) cNK cell populations at both the site of infection and in the spleen. Differences in cNK cell maturation may correlate with different cytokine profiles.

## Materials and Methods

### Mice

Female 6- to 8-week-old C57BL/6J mice (Jackson Labs) were used for all experiments. Animals were housed in specific pathogen-free conditions at the University of Wyoming Animal Facility.

### Ethics Statement

This study was carried out in strict accordance following the recommendations in the Guide for the Care and Use of Laboratory Animals of the National Institutes of Health. The protocol was approved by the Institutional Animal Care and Use Committee (IACUC) of the University of Wyoming (PHS/NIH/OLAW assurance number: A3216-01).

### *T. gondii* Parasites, Infections

Tachyzoites (tach.) of RH and RHΔ*cpsII* (*cps1-1*) strains (kindly provided by Dr. Bzik, Dartmouth College, NH, USA) were cultured by serial passage in human fetal lung fibroblast (MRC5) cell monolayers in complete DMEM (supplemented with 0.2 mM uracil for *cps1-1* strain). For mouse infections, parasites were purified by filtration through a 3.0-μm filter (Merck Millipore Ltd.) and washed with phosphate-buffered saline (PBS). Mice were infected intraperitoneally (i.p.) with 1 × 10^3^ RH tach. or 1 × 10^6^ *cps1-1* tach. The brains of the 5-week ME49-infected C57BL/6 mice were used as a source of ME49 cysts. Mice were infected i.p. with 10 ME49 cysts.

### Cell Surface and Intracellular Staining for Flow Cytometry

Peritoneal exudate cells (PECs) were harvested by washing peritoneum with 7 ml of ice cold PBS. Spleens were crushed through a 70-μm tissue strainer (VWR) and red blood cells lysed with RBC lysis buffer (Sigma). Single-cell suspensions were then plated at 1–2 × 10^6^ cells/well and stained for flow cytometry. For surface staining, cells were washed twice with 1× PBS and stained for viability in 1× PBS using Fixable Live/Dead aqua dye (Invitrogen) for 30 min. After washing with 1× PBS, surface staining was performed using antibodies diluted in stain wash buffer (SWB, 2% FBS in 1× PBS and EDTA) for 25 min on ice. For functional assays, total PECs and splenocytes were plated in wells coated with anti-NK1.1 (PK136, BioXcell). Cells were stimulated for 4 h in the presence of 1× protein transport inhibitor cocktail containing Brefeldin A/Monensin (eBiosciences) and anti-CD107a. After live dead and surface staining, cells were fixed and permeabilized for 1 h on ice (BD bioscience, Fix/Perm solution) followed by intracellular staining in permeabilization wash buffer with anti-IFNγ APC (eBiosciences) antibody. For proliferation assays, surface and intracellular staining was repeated as described above directly *ex vivo* without anti-NK1.1 restimulation and staining with anti-Ki67 APC (Biolegend) antibody. After washing with 1× PBS, cells were resuspended in 1× PBS and acquired using Guava easyCyte HT (Millipore). All samples were analyzed with FlowJo software (Tree Star).

### Antibodies

The following antibodies were purchased from Biolegend: CD3 BV510, CD49b (DX5) APC-Cy7, NKp46 BV421, CD11b PE, Ly49D PE, Streptavidin BV650, CD27 biotin, KLRG1 BV605, KLRG1 PerCP/Cy5.5, CD69 PerCP/Cy5.5, and Ki67 APC. The following antibodies were purchased from eBioscience: CD107a PerCP-eFluor710, Ly49I PE, CD94 FITC, IFNγ APC, and Ly49H PE-Cyanine7. The following antibodies were purchased from BD Biosciences: NKG2A/C/E BV605. The following antibodies were purchased from Miltenyl Biotec: NKG2D (CD314) biotin, Ly49D biotin, and Ly49D PerCP-Vio700. To determine viability Invitrogen Fixable live/dead Aqua stain was used.

### IFNγ Production by Isolated cNK Cells

Conventional natural killer cells were purified from the PECs and spleens harvested from 5-day-infected (*cps1-1*, RH, ME49) mice using EasySep Mouse NK cell isolation kit (STEMCELL Technologies). cNK cells were then plated at 0.2 × 10^6^ cNK cells in 0.2 ml of complete DMEM (10% FCS, penn/strep, amphotericin B) per well in polystyrene 96-well culture plate and cultured at 37°C for 24 h. After incubation, the cells underwent three freeze/thaw cycles and were then centrifuged at 14,000 rpm for 10 min. Supernatant was collected and used to measure IFNγ protein concentration by ELISA according to manufacturer’s protocol (Biolegend).

### Multiplex Cytokine Assays IL-12, IFNγ, IL-1β, IL-10, TNFα, IL-17A, IL-2, IL-21, and IL-15

Total PECs and splenocytes were obtained from 5-day-infected (*cps1-1*, RH, ME49) and control mice. Cells were plated at 2 × 10^6^ PECs and 5 × 10^6^ splenocytes per well in complete DMEM (10% FCS, penn/strep, amphotericin B) in polystyrene culture plate and cultured at 37°C for 24 h. After incubation, the cells underwent three freeze/thaw cycles and were then centrifuged at 14,000 rpm for 10 min. Supernatant was collected and used for cytokine determination. To obtain serum, whole blood from uninfected and 5-day-infected mice was harvested and incubated for 30 min at RT then centrifuged for 10 min at 14000 rpm. Serum was removed and used in cytokine assays. Multiplex cytokine assay was performed using MILLIPLEX MAP Mouse TH17 Magnetic Bead Panel (EMD MILLIPORE), according to the manufacturer’s instructions. Analysis was conducted in a Bio-plex MAGPIX multiplex reader. Mouse IL-12/23(p40) concentration was measured by ELISA according to manufacturer’s protocol (Biolegend).

### Real-time PCR for Parasite Burden

DNA was extracted from entire PECs and spleens harvested from infected mice using a Qiagen DNeasy Blood & Tissue Kit (Qiagen Sciences). Parasite DNA from 600 ng of PECs and 800 ng of splenic tissue DNA was amplified using primers specific for the *T. gondii* B1 gene (forward primer GGAACTGCATCCGTTCATG and reverse primer TCTTTAAAGCGTTCGTGGTC) at 20 pmol of each per reaction (Integrated DNA Technologies) by real-time fluorogenic PCR using SsoAdvanced™ Universal IT SYBR^®^ Green SMx (BIO-RAD) on a CFX Connect™ Real-Time System cycler (BIO-RAD). Parasite equivalents were determined by extrapolation from a standard curve.

### Statistical Analysis

Statistical analysis was performed using Prism 6.0f (GraphPad) or Microsoft Excel 2011. Significant differences were calculated using either unpaired Student’s *t*-test or analysis of variance (ANOVA). Significance is denoted where *p* < 0.05.

## Results

### cNK Cells Frequency and Absolute Cell Numbers

**Figure 1:**
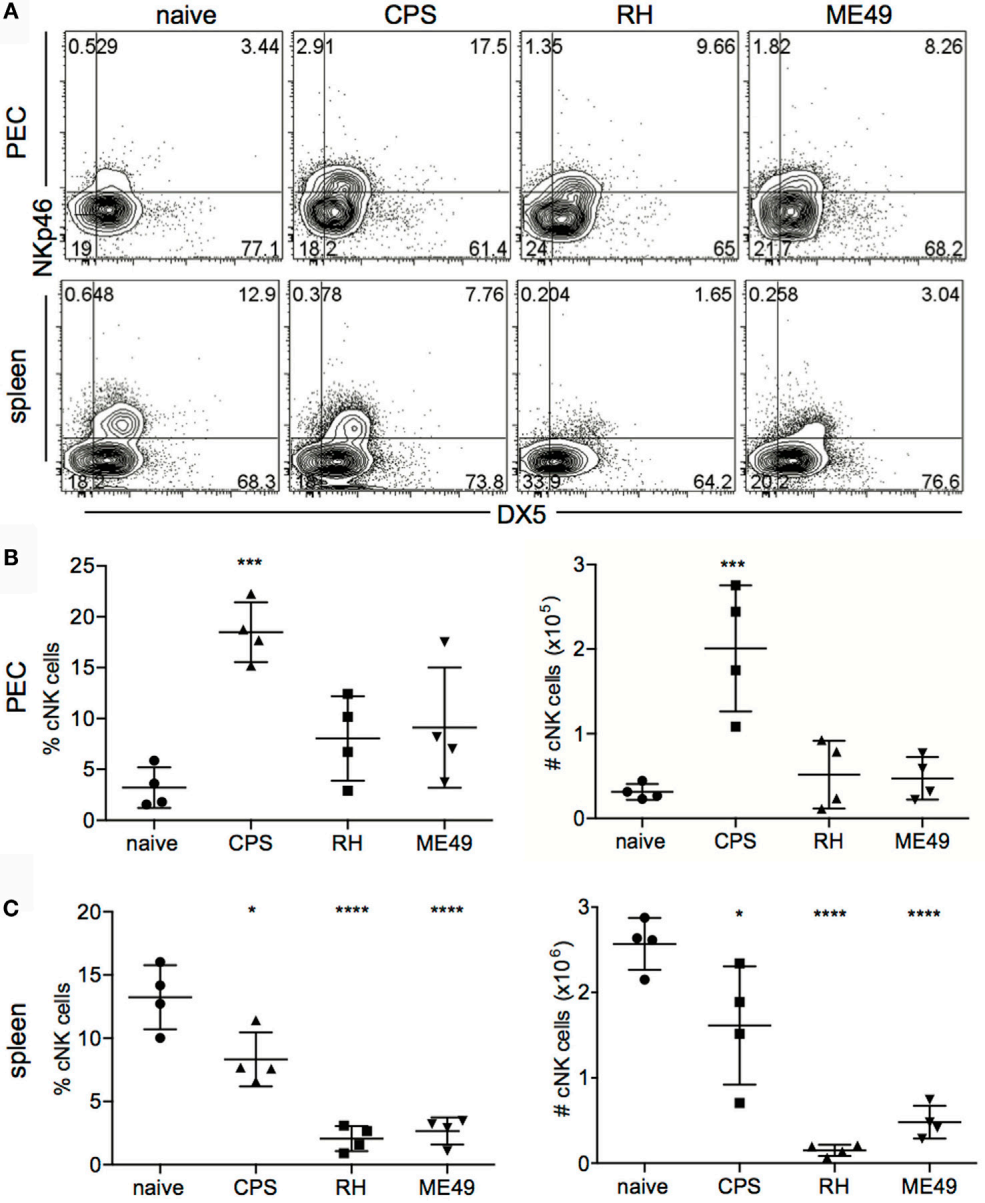
**cNK cell counts within mice peritoneal exudate cells and splenocytes after *T. gondii* infection**. WT female C57BL/6 mice were infected i.p. with 1 × 10^6^ *cps1-1*, 1 × 10^3^ RH tachyzoites or 10 brain cysts of ME49. Five days after infection, peritoneal exudate cells (PECs) and splenocytes were harvested and stained for analysis by flow cytometry. **(A)** cNK cells were identified as CD3−DX5+NKp46+ cells. Representative contour plots of cNK cell frequency in PEC (upper row) and spleen (lower row). **(B,C)** Scatter plots present the mean ± SD of cNK cell frequency (left graphs) and absolute cell number per organ (right graphs) calculated for individual mice in **(B)** PEC and **(C)** spleen. Data are representative of three experiments, *n* = 4 per experiment. Statistical differences are represented by *p < 0.05, ***p < 0.001, and ****p < 0.0001 in comparison with the control group (naive).

Different *T. gondii* strains can have differential effects on the activation of innate immune responses (3). Comparative analysis of cNK cell response to different *T. gondii* strains has not been performed. Therefore, we tested whether cNK cell responses to acute *T. gondii* infection were different depending on parasite strain. Mice were infected with Type I highly virulent RH, attenuated RH strain *cps1-1*, and Type II strain avirulent ME49, and cNK cell frequencies and numbers were measured at the site of infection (peritoneum, PEC) and spleen. Mice were harvested 5 days after i.p. infection, and cNK cells were enumerated. We chose day 5 as a time point because this is when we detect cNK cell responses after infection with all three parasite strains. In uninfected mice, cNK cells, identified as CD3−DX5+NKp46+, constituted on average 3.2 ± 2.0% of live PEC. Infection with *cps1-1* induced significant increase in cNK cell frequency (18.5 ± 2.9%) (Figure 1A). Five days post RH and ME49 infection, the frequency of cNK cells at the site of infection did not increase (Figure 1B). Absolute cNK cell numbers per PEC only increased after *cps1-1* infection and not after RH and ME49 infections (Figure 1B). cNK cell frequencies and numbers in spleen were different as compared with PEC. We found significant decreases in cNK cell frequency and absolute cell number after infection with all three strains in spleen (Figure 1C).

### cNK Cell IFNγ Production, Cytotoxicity, and Polyfunctionality

**Figure 2:**
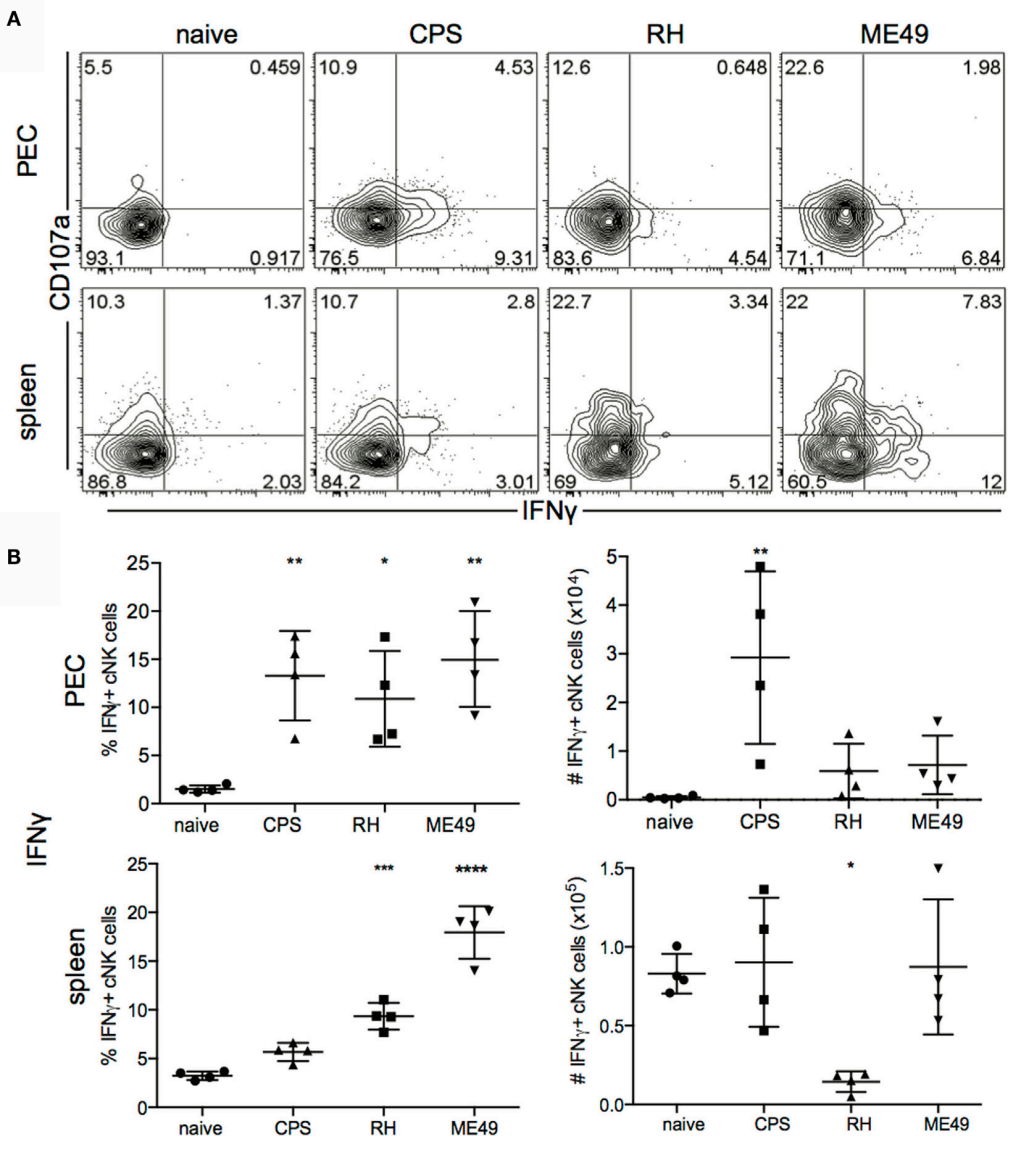

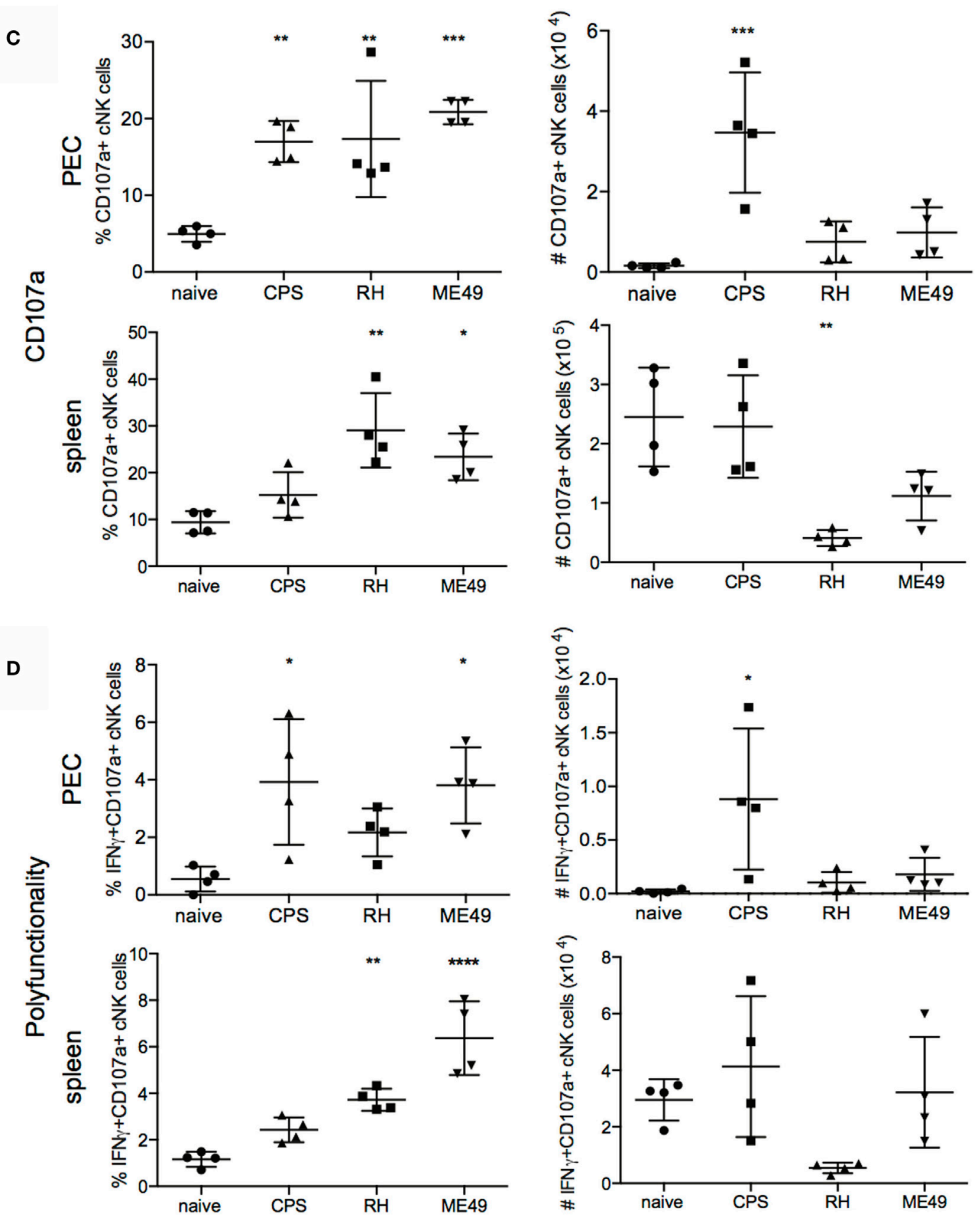
**IFNγ production and CD107a expression by cNK cells in mice after *T. gondii* infection**. Functionality of cNK cells was measured in PECs and spleens harvested from uninfected and *T. gondii*-infected mice 5 days post infection and stimulated *in vitro* with plate bound anti-NK1.1 for 4 h. Cells were stained for IFNγ and cytolytic activity with anti-CD107a. **(A)** Contour plots representing frequency of IFNγ+, CD107a+, and double positive polyfunctional IFNγ+CD107a+ cNK cells in PEC (upper row) and spleen (lower row). **(B)** Scatter plots present frequency (left graphs) and number (right graphs) of IFNγ+ cNK cells in individual mice in PEC and spleen. **(C)** Scatter plots present frequency (left graphs) and number (right graphs) of cytotoxic CD107a+ cNK cells in individual mice in PEC and spleen. **(D)** Scatter plots present frequency (left graphs) and number (right graphs) of IFNγ+CD107a+ cNK cells in individual mice in PEC and spleen. Data are representative of three experiments, *n* = 4 per experiment. Individual values are mean ± SD as shown. Statistical differences are represented by *p < 0.05, **p < 0.01, ***p < 0.001, and ****p < 0.0001 in comparison with the control group (naive).

Conventional natural killer cells provide protection against infection and cancer by producing IFNγ and cytolytic activity (27). Polyfunctional responses of cNK cells (2 or more functions per cell) are a positive prognostic marker for outcomes associated with certain infections (28, 29). T cell-independent cNK-produced IFNγ is critical for early control of infection (5, 30). The importance of cNK cytotoxicity is not well understood for *T. gondii* infection (10), and whether cNK cells develop a polyfunctional response to *T. gondii* is not known. Comparison of how different parasite strains impact cNK cell function has not been performed. Therefore, we measured cNK IFNγ, cytotoxicity as measured using the surrogate marker CD107 (31) and polyfunction (IFNγ+CD107a+) after infection with three different strains of *T. gondii* infection to address these questions. The frequency of IFNγ+ cNK cells in PEC increased after infections with all three strains (Figures 2A,B). However, the absolute number of IFNγ+ cNK cells per organ did not increase significantly after RH and ME49 infection (Figure 2B). Infection with the attenuated type I strain *cps1-1* induced an increase in both frequency and absolute number of IFNγ+ cNK cells. In spleen, the frequency of IFNγ-producing cNK cells increased after RH and ME49 infections (Figures 2A,B). In contrast, attenuated *cps1-1* parasites did not induce any increase in frequency of IFNγ-producing cNK cells in the spleen (Figures 2A,B). We did not detect an increase in absolute number in IFNγ+ cNK cells in the spleen. Interestingly, RH infection resulted in a significant decrease in the absolute numbers of cNK cells capable of producing IFNγ in the spleen (Figure 2B).

Previous studies demonstrate cNK cells can be cytotoxic against target cells stimulated with parasite components (9, 11). The level of cNK cell cytolytic activity in response to different parasite strains has not been measured. We next assessed cNK cell cytotoxicity using CD107a (LAMP-1, the surrogate marker of cytotoxicity) expressed by cNK cells. The pattern of cytotoxic cNK cell response was similar to cNK cell IFNγ response (Figure 2C). Cytotoxic CD107a+ cNK cells increased in frequency in peritoneum after infections with all three strains and in spleen after RH and ME49 infections only (Figure 2C). Absolute numbers of cytotoxic cNK cells also followed a similar to IFNγ-producing cNK cells pattern. CD107a+ cNK cells increased in number in the PEC after *cps1-1* infection, but not after RH and ME49 infection. In the spleen, RH and ME49 infection stimulated an increase in CD107a+ cNK cells. Interestingly, similar to IFNγ+ cNK cells, RH infection resulted in a significant decrease in CD107a+ cNK cell numbers, while ME49 induced less of a decrease and *cps1-1* infection did not alter cNK cytotoxic responses in the spleen (Figure 2C).

Polyfunctional cNK cell responses similar to polyfunctional T cell responses are an indicator of the quality and effectiveness of the immune response generated (28, 29). Whether *T. gondii* infection stimulates a polyfunctional cNK cell response is not well known. Polyfunctional responses of cNK cells were measured by cNK cells positive for both IFNγ and CD107a. Polyfunctional cNK cells increased in frequency in the PEC after *cps1-1* and ME49 infection (Figures 2A,D). RH infection did not stimulate a significant increase in polyfunctional cNK cell frequency in the PEC. Only infection with *cps1-1* increased the absolute number of polyfunctional cNK cells in the PEC compared with RH and ME49 (Figure 2D). In the spleen only RH and ME49 infections resulted in increased polyfunctional cNK cell frequencies (Figure 2). However, the absolute number of polyfunctional cNK cells in spleen did not significantly change after all infections.

**Figure 3:**
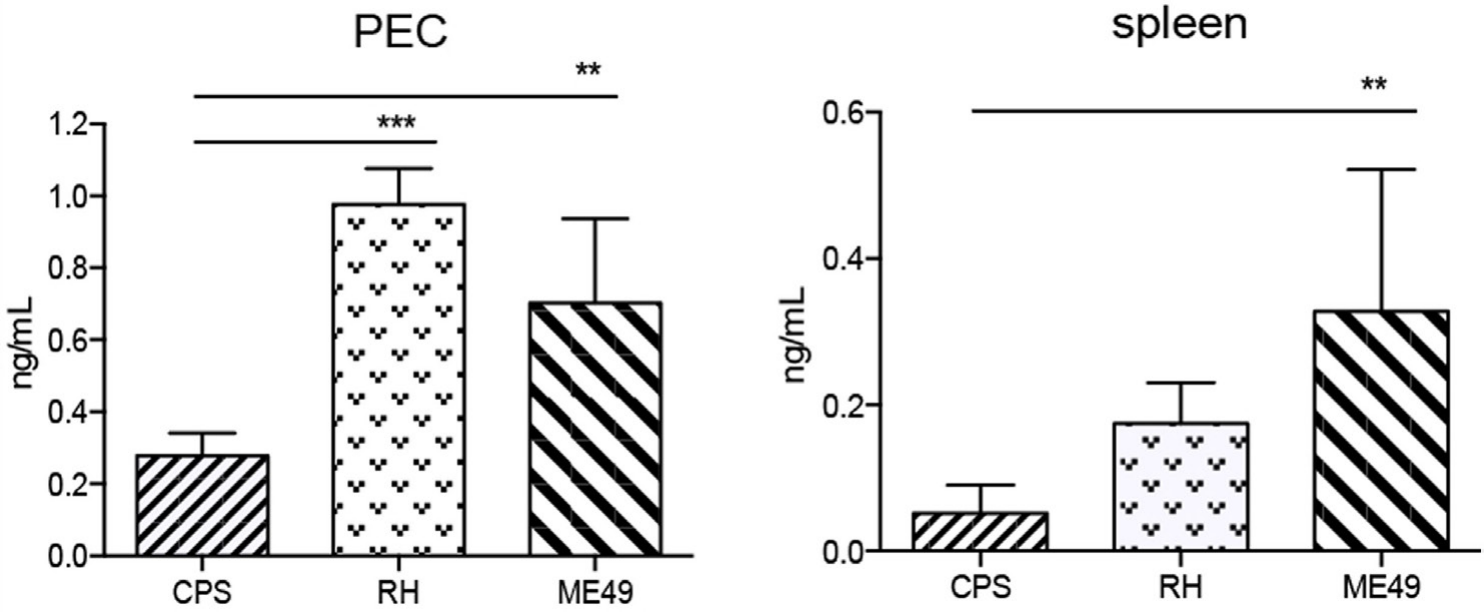
**Quantification of IFNγ production by cNK cells in mice after *T. gondii* infection**. cNK cells were purified from PECs and spleens 5-day-*T. gondii*-infected mice and cultured for 24 h to measure IFNγ protein in supernatants by ELISA. Bar graphs present concentration of IFNγ protein in mice in PEC and spleen. Data are representative of two experiments, *n* = 4 per experiment. Individual values shown are mean ± SD. Statistical differences are represented by *p < 0.05, and **p < 0.01.

The cNK cell function assays above were carried out using plate bound anti-NK1.1 to restimulate the cells for flow cytometry. NK1.1 cross-linking with anti-NK1.1 can stimulate cNK cells from naive mice. Therefore, to verify whether there were differences in the level of IFNγ produced by cNK cells at day 5 post infection with the different parasite strains, we purified cNK cells from infected animal PEC and spleen by negative selection. Normalized numbers of cNK cells from their respective tissue source were cultured, and IFNγ was quantified in supernatants by ELISA. Peritoneal cNK cells from RH-infected mice produced the highest quantity of IFNγ, followed by cNK cells from ME49-infected mice and the lowest quantity of IFNγ was produced by cNK cells isolated from *cps1-1*-infected mice (Figure 3). Splenic cNK cells isolated from ME49- and RH-infected mice produced IFNγ, but less than peritoneal cNK cells. As expected, splenic cNK cells from *cps1-1* mice produced barely detectable amounts of IFNγ.

### Activating and Inhibitory Receptors Expression on cNK Cells

**Figure 4:**
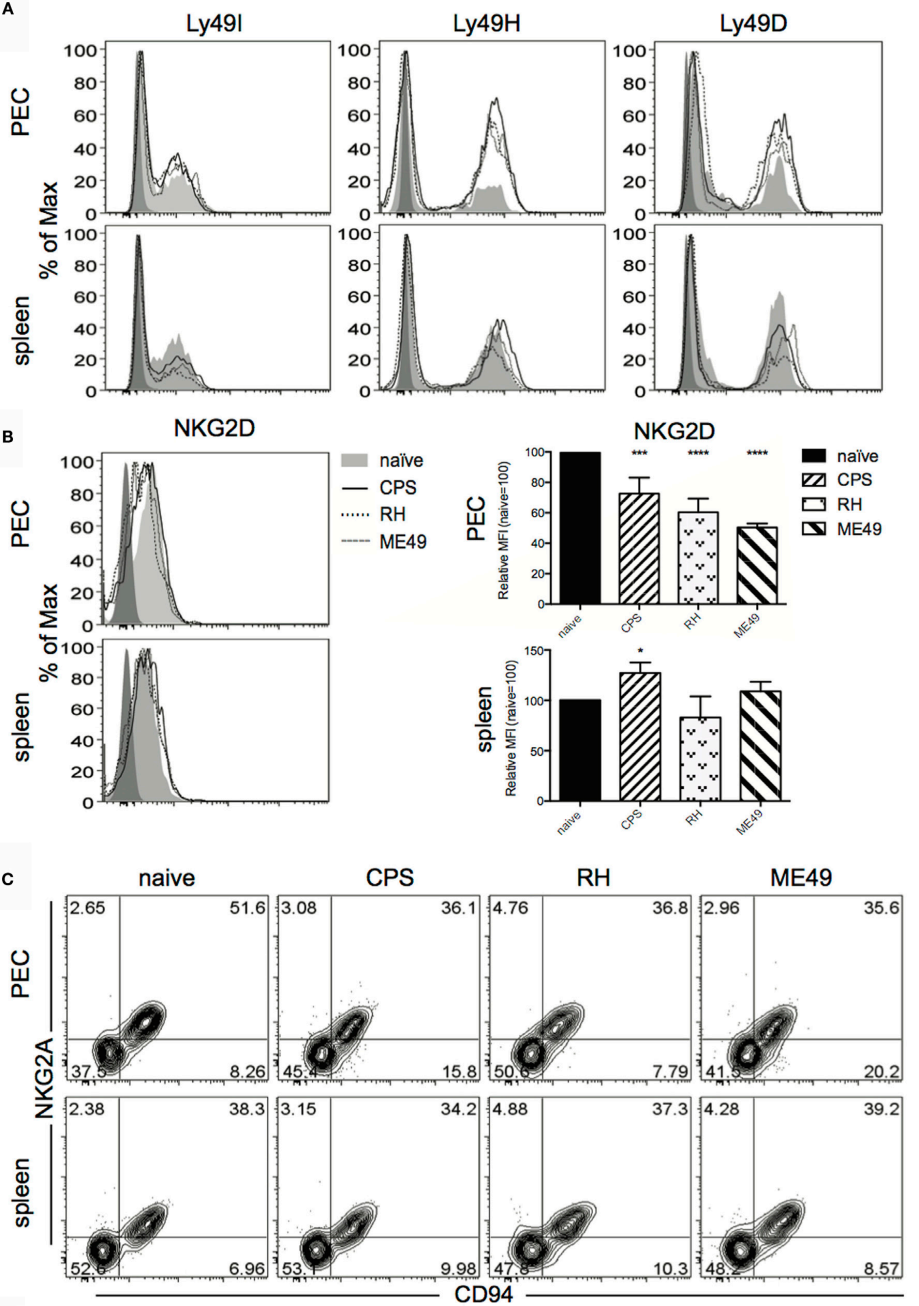

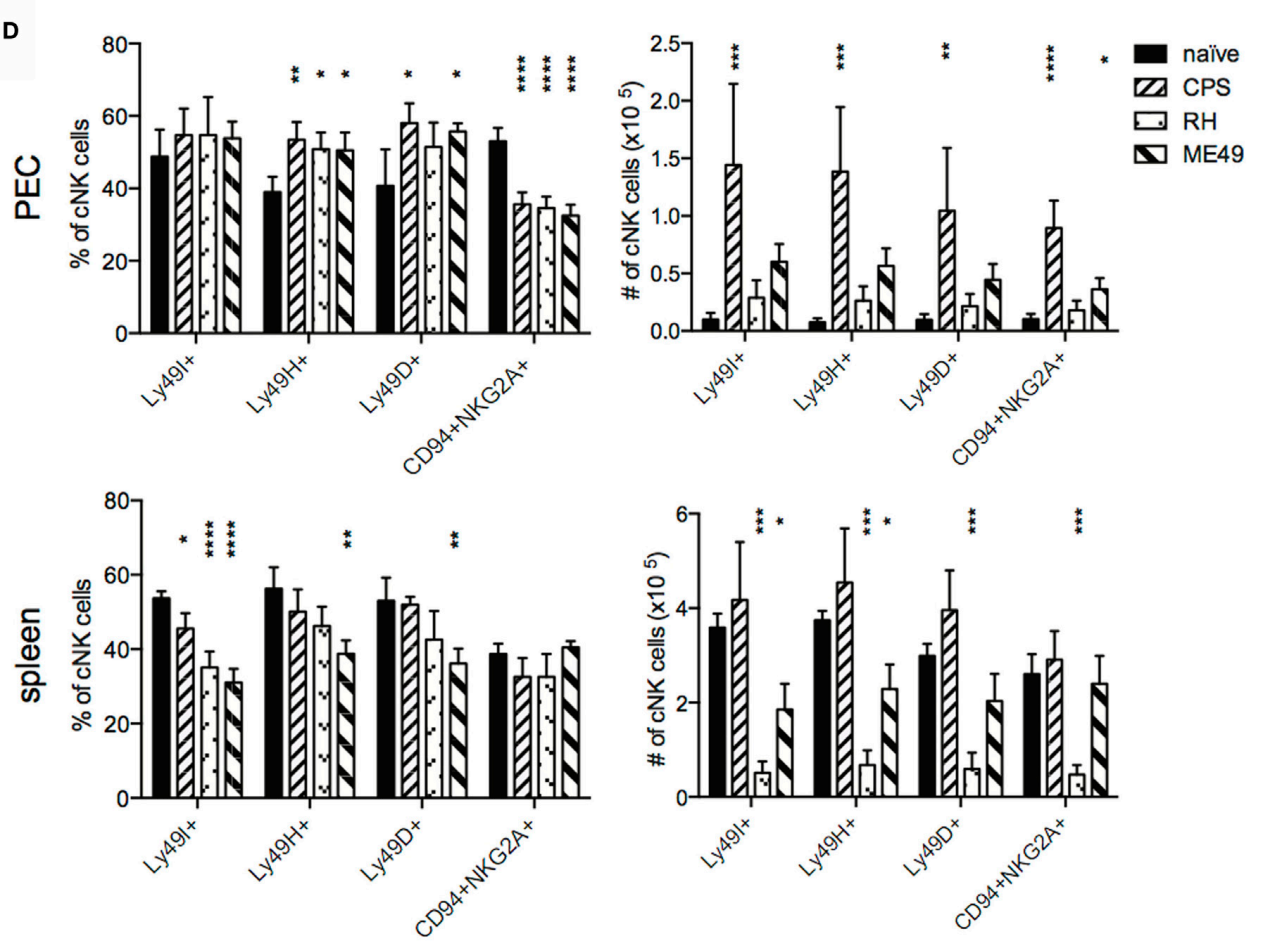
**Activating and inhibitory receptor expression by cNK cells within mice peritoneal exudate cells and splenocytes after *T. gondii* infection**. PECs and splenocytes isolated from uninfected and 5 day-*T. gondii*-infected mice were stained for analysis by flow cytometry for Ly49I, Ly49H, Ly49D, NKG2D, NKG2A, and CD94 expression on cNK cells (CD3−DX5+NKp46+). **(A)** Histograms present frequency of receptor positive and expression levels of Ly49I, Ly49H, and Ly49D on cNK cells in PEC and spleen. **(B)** Histograms present frequency of receptor positive and expression levels of NKG2D on cNK cells in PEC and spleen. Bar graphs present relative mean fluorescence intensity of NKG2D levels on cNK cells in PEC and spleen. **(C)** Representative contour plots of cNK cells gated for CD94/NKG2A expression in PEC and spleen. **(D)** Scatter plots present frequency and number of Ly49I+, Ly49H+, Ly49D+, and CD94+NKG2A+ NK cells in individual mice in PEC and spleen. Data are representative of three experiments, *n* = 4 per experiment. Individual values are mean ± SD as shown. Statistical differences are represented by *p < 0.05, **p < 0.01, ***p < 0.001, and ****p < 0.0001 in comparison with the control group (naive).

Activating and inhibitory receptors tune how cNK cells respond to infection and are expressed in a stochastic array on cells (16). Stochastic expression can give rise to a bulk cNK population composed of many different subsets or subpopulations (16). There can be two phases of cNK cell responses, one that is cytokine dependent and the second being dependent upon engagement of activating receptor with cognate ligand (32, 33). MCMV infection is a good example of this biphasic response. cNK cells respond to Type I IFNs and IL-12 to initiate proliferative burst of cNK cells followed by selection of a specific cNK cell population where the viral protein m157 is recognized by the activating receptor Ly49H, thus promoting the survival and enrichment of this population (33, 34). Currently, whether a dominant cNK cell population arises to control *T. gondii* infection is not known. In addition, it is not clear how infections with different *T. gondii* strains change bulk cNK cell subpopulation composition based on activating and inhibitory receptor expression. Therefore, cNK cells were analyzed for expression of activating and inhibitory receptors in order to characterize changes in subpopulation composition after infection with three different *T. gondii* strains. Activating receptors (Ly49H, Ly49D, NKG2D) and inhibitory receptors (Ly49I, CD94/NKG2A) expressed by cNK cells in PEC and in spleen were measured 5 days after infection (Figure 4). In PEC, the frequency of activating receptor Ly49H and Ly49D expressing cNK cells increased after infection with *cps1-1*, RH, and ME49 compared with naive controls (Figures 4A,D). The expression level of activating receptor NKG2D and the frequency of inhibitory receptor CD94/NKG2A cNK cells were reduced when compared with naive controls after infection with each strain of the parasite (Figures 4B–D). Inhibitory receptor Ly49I expressing cNK cells did not change in frequency. Absolute numbers in PEC revealed that among all three strains, *cps1-1* infection stimulated the greatest increase in cNK subsets expressing activating receptors Ly49H and Ly49D, as wells as inhibitory receptors Ly49I and CD94/NKG2A (Figure 4D). The fold increase in absolute number of Ly49I+, Ly49H+, Ly49D+ cNK cells was greater than in CD94+ NKG2A+ cNK cells. In spleen, the most significant difference was a reduction in frequency and absolute number of cNK cells in a graded manner with increasing virulence of the strain (Figure 4D). Infection with *cps1-1* did not stimulate a significant change in cNK cell subpopulation composition in the spleen (Figure 4D).

### The Phenotype of IFNγ-Producing cNK Cells

**Figure 5:**
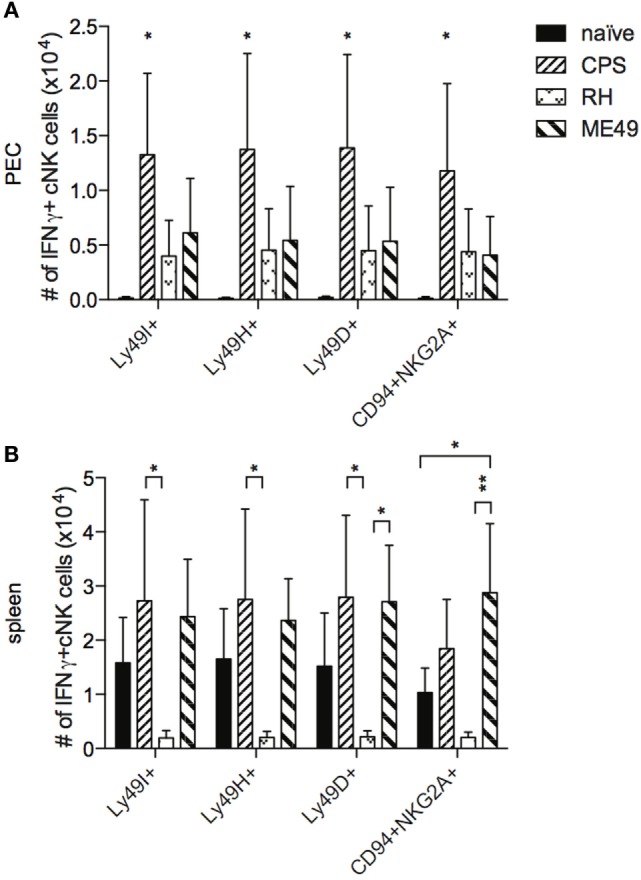
**IFNγ production by distinct populations of cNK cells after *T. gondii* infection**. PECs and splenocytes from uninfected and infected mice were restimulated *in vitro* with plate bound anti-NK1.1 for 4 h, then stained for surface receptors, fixed, and subsequently stained for intracellular IFNγ. **(A,B)** cNK cells that were IFNγ+ were then gated for cNK cell receptors Ly49I, Ly49H, Ly49D, and CD94/NKG2A. Bar graphs present absolute cell number of each population per **(A)** PEC and **(B)** spleen. Data are representative of three experiments, *n* = 4 per experiment. Individual values are mean ± SD as shown. Statistical differences are represented by *p < 0.05 and **p < 0.01, in comparison with the control naive group **(A)** and CPS or ME49 group **(B)**.

Measuring changes in cNK cell subpopulations by activating or inhibitory receptor expression may not provide enough resolution to identify dominant cell populations responding to infection as shown in Figure 4. To better assess cNK cell subpopulation profiles, we analyzed IFNγ+ cNK cells for receptor expression. cNK cells were stimulated *ex vivo* after 5 days of infection with anti-NK1.1 and stained intracellularly for IFNγ production. cNK cells were surface stained for activating (Ly49H and Ly49D) and inhibitory (Ly49I and CD94/NKG2A) receptors and analysis was performed on IFNγ+ cNK cells. In PEC, the numbers of IFNγ+ cNK cells expressing inhibitory receptors Ly49I or CD94/NKG2A and activating receptors Ly49H, Ly49D increased significantly after *cps1-1* infection and not significantly after RH and ME49 infections (Figure 5A). In spleen, *cps1-1* and ME49 infection had little impact on the receptor phenotype of IFNγ+ cNK cells. After RH infection, there was a reduction in absolute numbers of IFNγ+ cNK cells positive for each receptor (Figure 5B).

### cNK Cell Maturation

**Figure 6:**
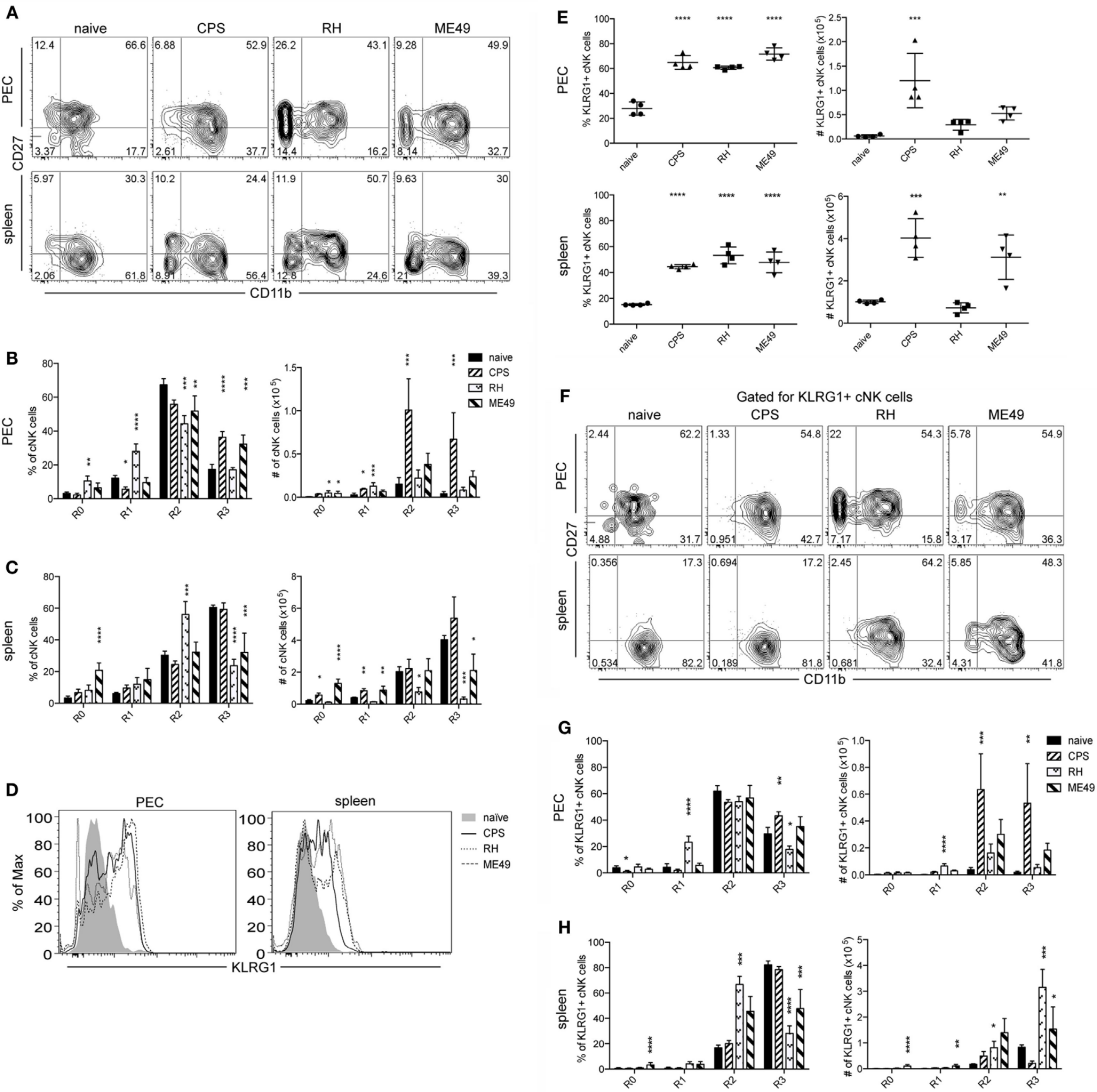
**cNK cell maturation after *T. gondii* infection in mice**. PECs and splenocytes from uninfected and infected mice were harvested and stained for CD27, CD11b, and KLRG1 to assay for cNK cell maturation. **(A)** Representative contour plots of cNK cells gated for CD11b and CD27 expression in PEC and spleen are presented. **(B)** Bar graphs present frequency and number of CD27−CD11b− (R0), CD27+CD11b− (R1), CD27+CD11b+ (R2), and CD27−CD11b+ (R3) cNK cells in **(B)** PEC and **(C)** spleen. **(D)** Representative histograms present KLRG1 expression level on cNK cells in PEC and spleen. **(E)** Scatter plots present frequency and number of KLRG1+ cNK cells in individual mice in PEC and spleen. **(F)** Representative contour plots of KLRG1+ cNK cells gated for CD11b and CD27 expression in PEC and spleen are presented. **(G)** Bar graphs present frequency and number of CD27−CD11b− (R0), CD27+CD11b− (R1), CD27+CD11b+ (R2), and CD27−CD11b+ (R3) cNK cells in **(G)** PEC and **(H)** spleen. Data are representative of three experiments, *n* = 4 per experiment. Individual values are mean ± SD as shown. Statistical differences are represented by *p < 0.05, **p < 0.01, ***p < 0.001, and ****p < 0.0001 in comparison with the control group (naive).

Conventional natural killer cells function during infection can be dependent on cNK cell maturation (26). cNK cells move through four distinct maturation steps marked by changes in expression of CD27 and CD11b (24, 25). Stage 1 immature cNK cells are CD27−CD11b−, followed by stage 2 CD27+CD11b−, stage 3 moderately mature CD27+CD11b+ and stage 4 highly mature CD27−CD11b+ (25). Fully mature cNK cells acquire full functional competence, express a full complement of activating and inhibitory receptors, reduce their proliferative potential, and increase their migratory capacity (24). The cNK cell maturation profile and its correlation with activation and function during *T. gondii* infection are unknown. cNK maturation was measured 5 days after infection with *cps1-1*, RH, and ME49. As shown in Figure 6, cNK cell maturation status was determined based on expression of CD27 and CD11b as R0 (CD27−CD11b−), R1 (CD27+CD11b−), R2 (CD27+CD11b+), and R3 (CD27+CD11b+). In naive mice, the majority of cNK cells were represented by R2 subpopulation in peritoneum and R3 in spleen (Figure 6). Infection with *cps1-1* and ME49 parasites resulted in increased mature cNK cells in the PEC compared with naive controls (Figures 6A,B). The frequency and number of mature (R3) cNK cells in the PEC of RH-infected animals was significantly decreased compared with *cps1-1* and ME49 (Figures 6A,B). RH infection appeared to stimulate a significant increase in frequency and absolute number of R0 and R1 immature cNK cells (Figures 6A,B). R0 immature cNK cells also were increased after ME49 infection, but not to the extent of RH-infected animals in the PEC (Figures 6A,B). The pattern was different in the spleen. Infection with either of three parasite strains, in particular with ME49, induced increase in R0 and R1 cNK cells in spleen. R2 subpopulation in the spleen (Figures 6A,C) was enriched in frequency after RH infection. Interestingly, unlike after *cps1-1* infection, highly mature R3 cNK cells were decreased after both RH and ME49 infections in spleen (Figures 6A,C).

Mature and activated cNK cells upregulate their KLRG1 expression, and KLRG1 is considered to be a marker of terminal maturation (35). The frequencies of KLRG1+ cNK cells increased in both site of infection and in the periphery in a response to all infections (Figures 6D,E). The absolute numbers of KLRG1+ cNK cells in PEC increased in response to *cps1-1*, but not to RH and ME49 infection. Absolute numbers of KLRG1+ cNK cells increased in spleen after *cps1-1* and ME49 infection compared with RH, where numbers were comparable to naive controls (Figure 6E).

Since we observed the increase in the immature CD11b− cNK cell subsets and, simultaneously, an increase in highly mature KLRG1+ cNK cells after RH and ME49 infections, we, next, assessed the distribution of KLRG1+ cNK cells between CD11b− (R0, R1) and CD11b+ (R2, R3) subsets. We found in *cps1-1*-infected mice, KLRG1+ cNK cells had an expected distribution within mature CD11b+ subsets, similarly to uninfected mice (Figures 6F–H). Unlike *cps1-1* infection, after RH and ME49 infection, KLRG1 on cNK cells was more widely expressed across all maturation subsets. KLRG1 was highly expressed by immature CD11b− subsets and, in particular, within R1 compartment in PEC after RH infection and within R0 and R1 in spleen after ME49 infection.

### cNK Cell Activation and Proliferation after *T. gondii* Infection

**Figure 7:**
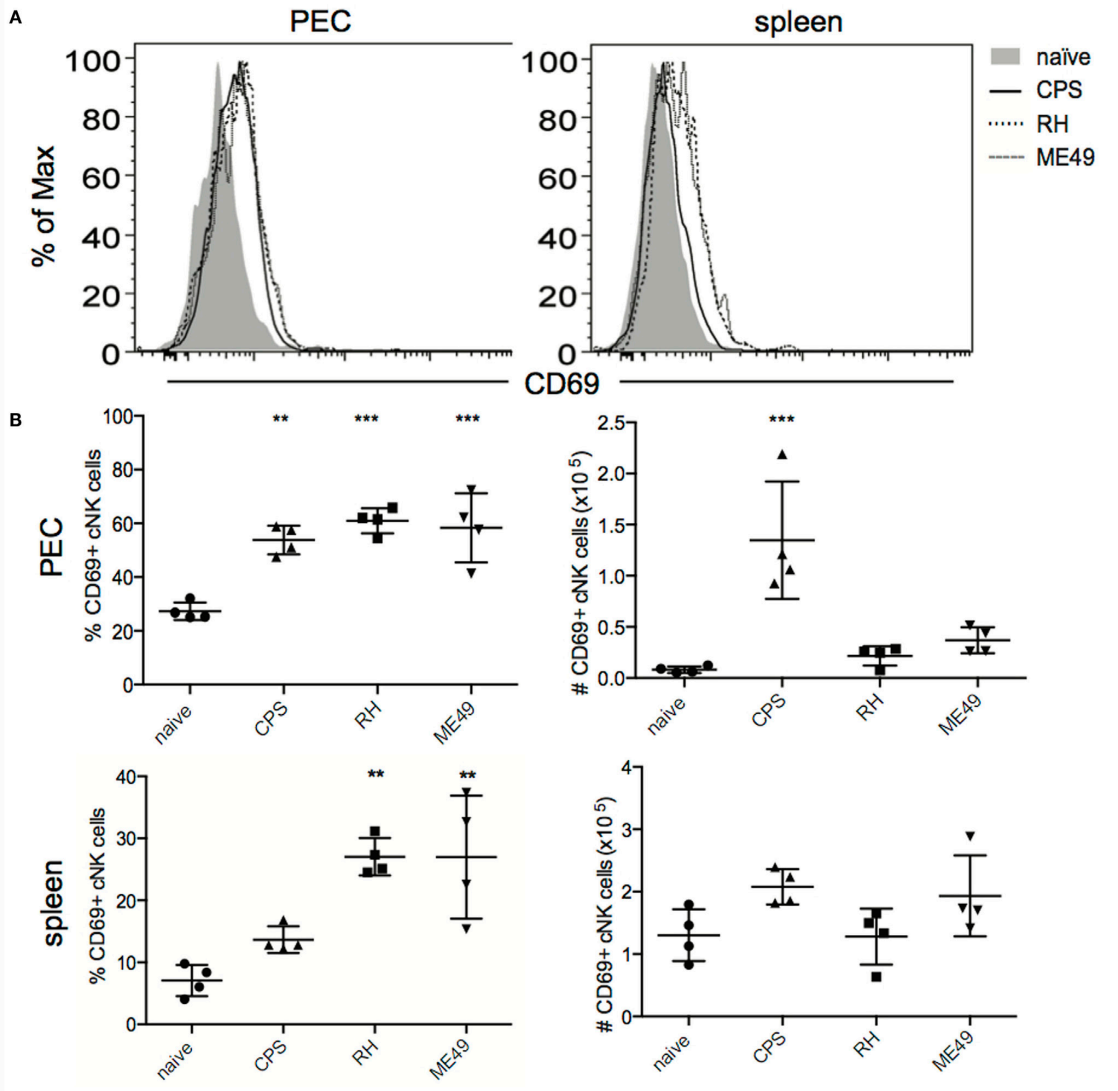

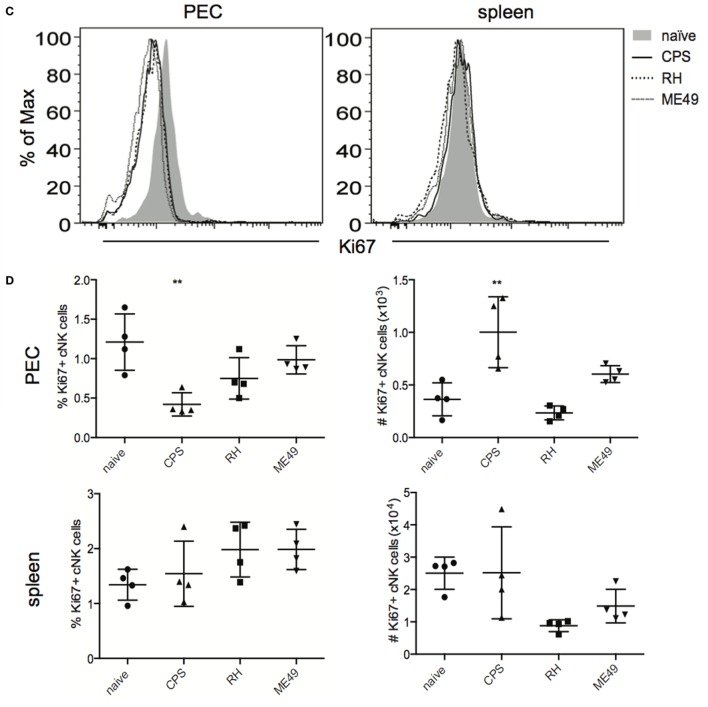
**cNK cell activation and proliferation after *T. gondii* infection**. cNK cell activation was measured by staining for CD69 expression on cNK cells (CD3−DX5+NKp46+) directly *ex vivo*. Proliferation of cNK cells was measured by intracellular staining for Ki67 directly *ex vivo*. **(A)** Representative histograms present CD69 expression level on cNK cell in PEC and spleen. **(B)** Scatter plots present frequency and number of CD69+ cNK cells in individual mice in PEC and spleen. **(C)** Representative histograms present Ki67 expression level in cNK cells in PEC and spleen. **(D)** Scatter plots present frequency and number of Ki67+ cNK cells in individual mice in PEC and spleen. Data are representative of three experiments, *n* = 4 per experiment. Individual values are mean ± SD as shown. Statistical differences are represented by *p < 0.05, **p < 0.01, ***p < 0.001, and ****p < 0.0001 in comparison with the control group (naive).

CD69 is upregulated on activated cNK cells and is an indicator of their migratory capacity (36, 37). CD69 expression has also been shown to have a regulatory function for cNK cells (38, 39). To further define how cNK cells respond to *T. gondii* infection and to compare how different parasite strains affect cNK cell activation and migration, we measured CD69 expression on cNK cells directly *ex vivo* without restimulation. The expression of CD69 (Figures 7A,B) correlated well with cNK cell function and maturation (Figures 2 and 6). The frequency of CD69+ cNK cells was increased after infection with all the strains in PEC and after RH and ME49 infections in spleen (Figures 7A,B). Absolute numbers of CD69+ cNK were increased in the PEC only after *cps1-1* infection and not after RH or ME49. Absolute numbers of CD69+ cNK cells in the spleen did not change after infection with all three strains.

Conventional natural killer cell proliferation was measured after *T. gondii* infection by intracellular staining for nuclear Ki67, a marker of cell cycle progression and a surrogate marker of cellular proliferation (40, 41). cNK cells did not appear to undergo proliferation at 5 days post infection in the PEC or spleen with any of the parasite strains tested (Figures 7C,D). Proliferative burst of cNK cells may occur earlier than 5 days post infection.

### The Levels of Cytokines in Mice Peritoneum, Spleen and Plasma

**Figure 8:**
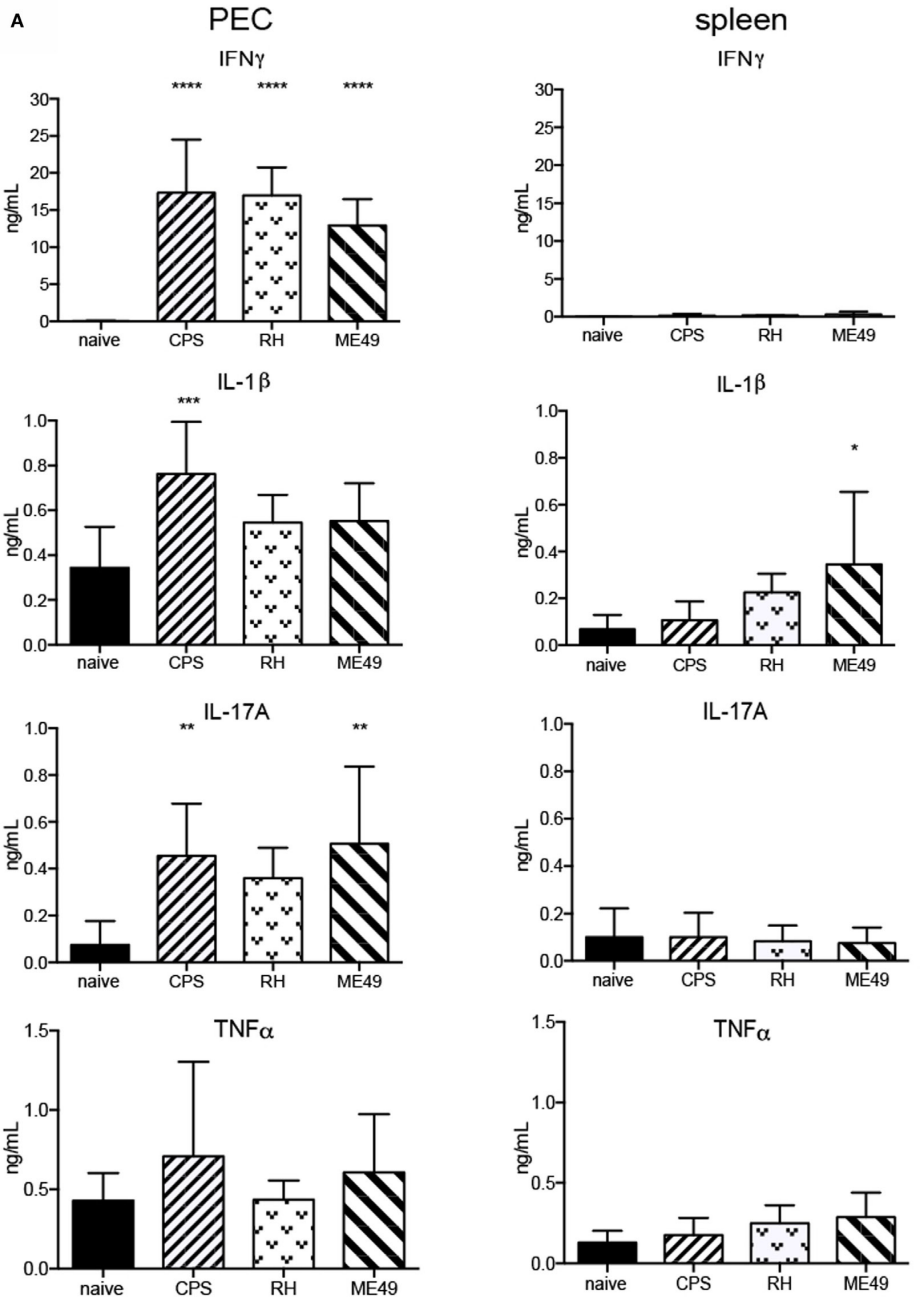

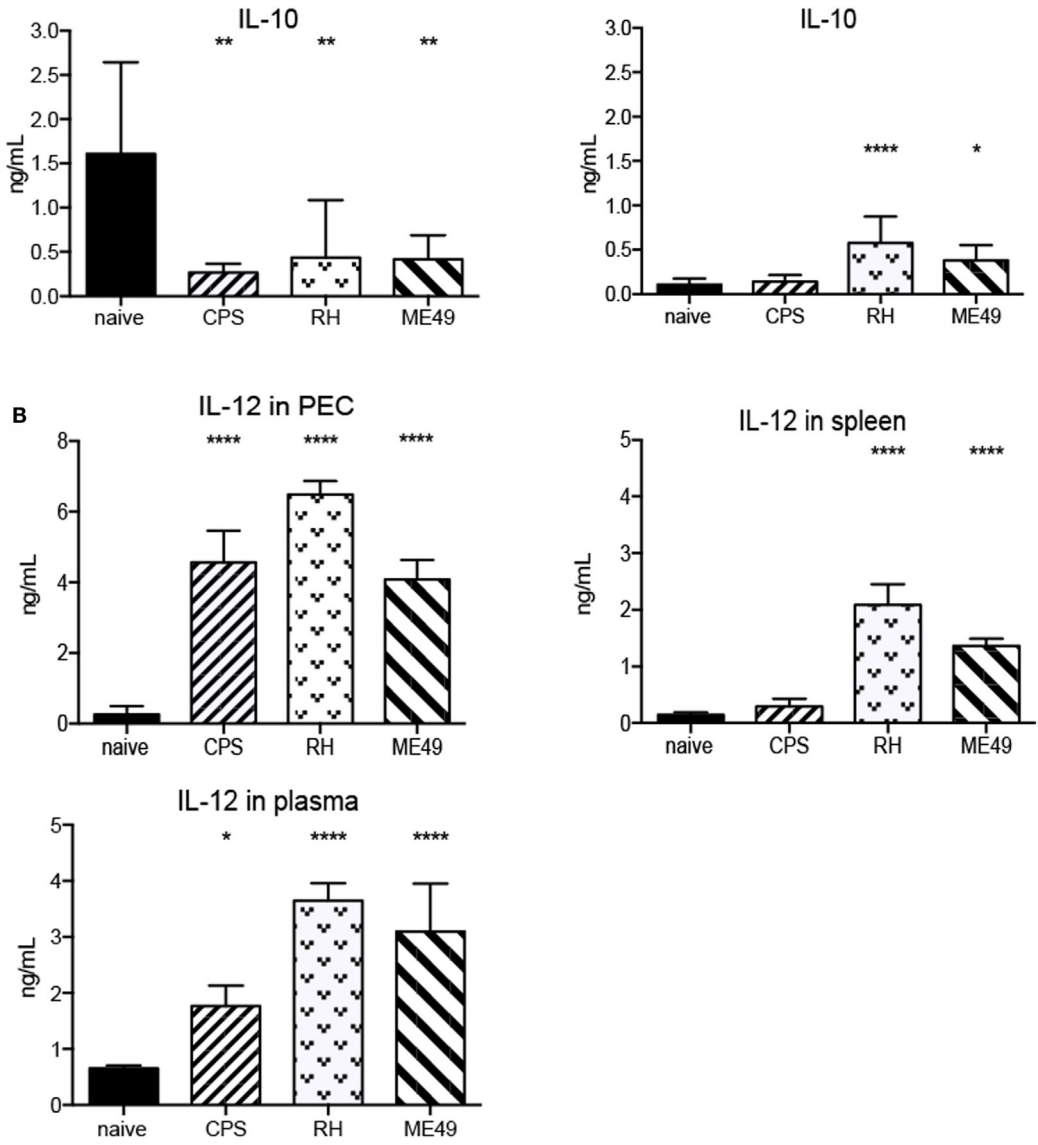
**Cytokine levels in mice peritoneum, spleen, and plasma after *T. gondii* infection**. Multiplex cytokine analysis was performed with PEC and splenocytes from naive and 5-day-infected animals. PECs and splenocytes were harvested and plated for 24 h. The concentrations of IFNγ, IL-1β, IL-10, TNFα, and IL-17A were simultaneously measured by multiplex assay in cultured cell supernatants. **(A)** Bar graphs present combined data from two separate experiments with an *n* = 4 animals per infectious parasite strain per experiment. **(B)** Bar graphs present combined data from two separate experiments with an *n* = 4 animals per infectious parasite strain per experiment. Individual values shown are mean ± SD. Serum was harvested from naive and infected animals. Supernatant from PEC, splenocytes, and serum were assayed for IL-12p40 by ELISA. Individual values shown are mean ± SD. Statistical differences are represented by *p < 0.05, **p < 0.01, ***p < 0.001, and ****p < 0.0001 in comparison with the control groups (naive).

The activation of cNK cells is highly dependent upon the inflammatory cytokine milieu present during infection and specifically upon IL-12 (6, 42). The virulence of *T. gondii* strain types is associated with their ability to induce quantitatively and qualitatively different cytokine production (3). How the cytokine milieu correlates with cNK cell responses to infection with different *T. gondii* strains is not known. To address this question, the concentration of proinflammatory and immunoregulatory cytokines produced at the site of infection, in spleen and serum, were tested using a multiplex approach (43). Additionally, ELISA analysis was performed to measure IL-12p40 at these different sites. Compared with naive animals, the level of IFNγ, IL-1β, IL-17A, and IL-12 was increased at 5 days post infection in PEC (Figure 8). The level of immunoregulatory cytokine IL-10 was decreased in PEC after infections with all three strains (Figure 8A). The concentrations of all cytokines in spleen were lower than at the site of infection. The level of IFNγ was similar after all infections (Figure 8); IL-1β was the highest in PEC after *cps1-1* and in spleen after ME49; IL-17A was increased after *cps1-1* and ME49 in PEC; IL-10 was increased after RH and ME49 in spleen. The concentrations of IL-2, IL-15, and IL-21 were beyond detection minimum (data not shown). ELISA for IL-12p40 indicated that RH infection induced the highest increase in production of this cytokine in PEC, spleen, and serum (Figure 8B). Infection with ME49 strain showed similar IL-12p40 production profiles as the RH strain. *Cps1-1* infection induced increase of the peritoneal and serum, but not splenic IL-12p40 (Figure 8B).

### *T. gondii* Parasite Burden in Mice Peritoneum and Spleen

**Figure 9:**
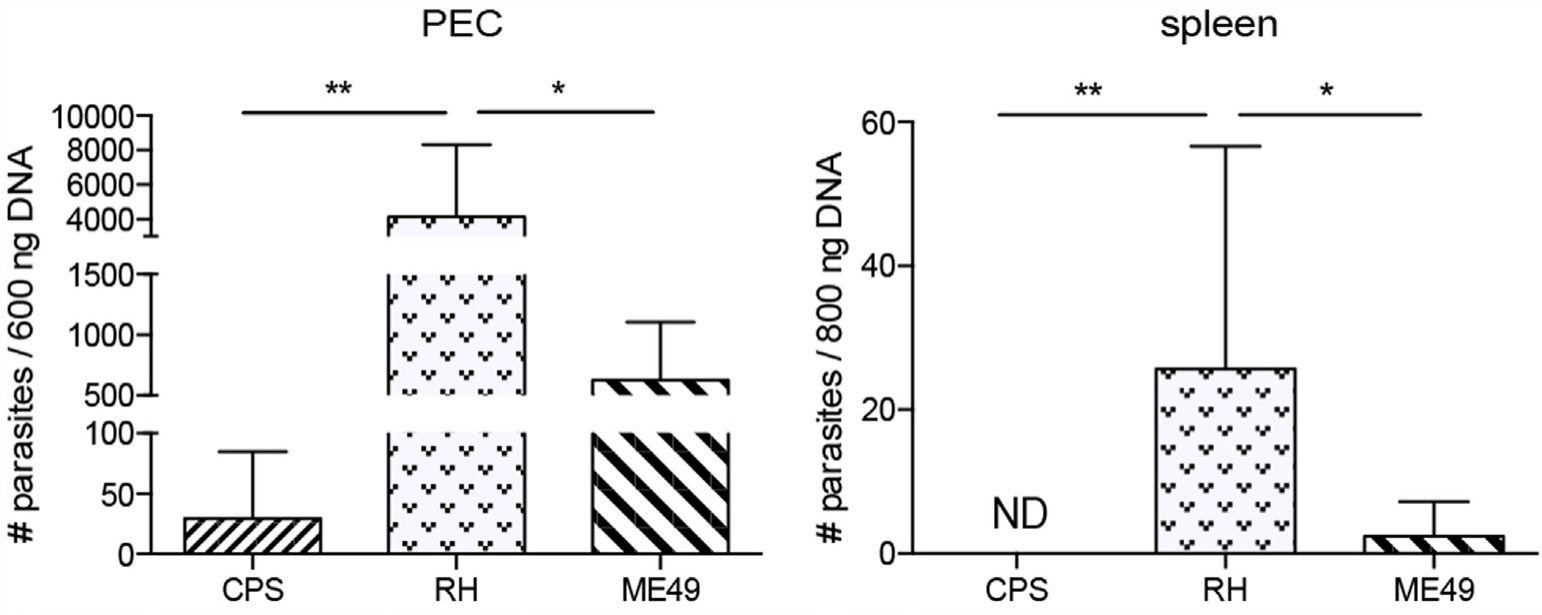
**The parasite numbers in mice peritoneum and spleen 5 days after *T. gondii* infection**. Real-time semi-qPCR was performed to amplify *T. gondii*-specific B1 gene present in the PECs and spleens harvested from 5-day-*T. gondii*-infected mice. Parasite equivalents were determined by extrapolation from a standard curve. Bar graphs present combined data from three separate experiments with an *n* = 4 animals per infectious parasite strain per experiment. Individual values shown are mean ± SD.

Differences observed in cNK cell responses measured in this manuscript could be due to the amount of parasites present in each of the tissues. Although we used common dosages, 1 × 10^6^ *cps1-1*, 1 × 10^3^ RH tach., and 10 ME49 cysts, the initial infectious dose was not normalized between strains. Therefore, it is important to measure parasite burdens in each tissue. Parasite numbers were quantified using real-time PCR for parasite-specific B1 gene in PEC and in spleen 5 days after infection. As expected, attenuated *cps1-1* parasites that are unable to proliferate in the absence of uracil were found at very low levels in PEC and not detected in spleen (Figure 9). In contrast, higher numbers of RH and ME49 parasites were detected in both PEC and spleen. The parasite loads were higher after infection with virulent type I than avirulent type II strain.

## Discussion

Conventional natural killer cells are critical for early immunity to *T. gondii* infection (5, 30). How infection with *T. gondii* strains of different virulence affects cNK cell biology and their ability to respond is not known. Here, a comprehensive assessment of cell number, subpopulation composition based on surface receptor expression, function, phenotype of functional cells, maturation, activation, and proliferation of cNK cells was performed to determine how different *T. gondii* strains impact their response during acute infection. The findings demonstrate cNK cells were activated and functional at the site of infection (PEC) and in spleen in a graded manner after infection with RH, ME49, and *cps1-1* parasites. cNK cells were not proliferating at day 5 post infection. A dominant responding cNK cell phenotype based on activating and inhibitory receptor expression was not observed after infection with any of the strains. Maturation of cNK cells after both RH and ME49 infections was also reduced, and immature stages of cNK cells were more pronounced than after *cps1-1* infection. KLRG1 expression on cNK cells was induced by all infections with KLRG1 expressed on both mature and immature cNK cells after RH and ME49 infections. In contrast, after *cps1-1* infection, only mature CD11b+ cNK cells expressed KLRG1. In spleen, the frequency of CD69+ cNK cells was higher on those from RH- and ME49-infected animals compared with *cps1-1*, which may indicate higher level of activation-induced cell death (39, 44) and explain the loss of the mature cNK cell population in RH and ME49 infections. In both PEC and spleen, the highest parasite burdens were detected after infection with RH strain, followed by ME49 strain. Parasites were detected in PEC only and not in spleen after *cps1-1* infection. Taken together, the data suggest that, as parasite load increases, cNK cell responses may be negatively affected giving rise to a defective cNK cell compartment.

### cNK Cell Number after *T. gondii* Infection

The presence of *T. gondii* parasites appears to negatively impact splenic cNK cell counts in a graded manner, after intraperitoneal infection. Whether this is also dependent upon parasite virulence is not clear. We observed decrease in splenic cNK cell frequency and numbers 5 days after both type I RH and type II ME49 in comparison to the *cps1-1* infection. Contraction of cNK cells has been shown after *P. yoelii* infection in mice (45), in *Plasmodium falciparum*-infected patients (46), after MCMV infection (47), and in a Con A-induced hepatitis model (48). This decrease in cNK cell counts could potentially be explained by the migration of splenic cNK cells to other sites, in particular, to the site of infection. Our observations may support this idea because cNK cells did not proliferate 5 days after the infection in PEC or spleen. cNK cells may proliferate earlier or later in response to *T. gondii* infection. Infection also increased mature KLRG1+ cNK cell numbers adding support for this hypothesis because mature cNK cells are more capable of migration than proliferation (49). Interestingly, i.p. IFNγ treatment mobilizes cNK cells from spleen and bone marrow into circulation and promotes cNK cell accumulation, but not proliferation in the peritoneum and tumor metastatic sites (48). Despite this possibility, there was no increase in cNK cell numbers in peritoneum after i.p. infection with replicating parasites, in contrast to non-replicating *cps1-1* strain. Another explanation to the above observation could be the death of cNK cells induced by the infection with virulent parasites. Indeed, depending on the parasite virulence, *T. gondii* infection triggers activation of the immunoregulatory mechanisms (50–52). One factor involved in immune regulation is CD69 (39, 44). CD69 has been shown to prevent immunopathology caused by *Listeria monocytogenes* infection (44). Additionally, CD69 deficient animals were better protected against vaccinia virus and had increased cNK cell numbers due to the decreased cell death rate (39). Thus, increased CD69+ cNK cells after RH and ME49 infection could be indicative of increased cNK cell apoptosis. In summary, both cNK cell migration and cell death due to inflammation may contribute to the decrease of cNK cell counts in spleen after infection with virulent *T. gondii* strains.

### cNK Cell Function

Different strains of *T. gondii* trigger differing levels of cNK cell activation in PEC and in spleen. cNK cells are a source of IFNγ during acute *T. gondii* infection (8, 30). cNK cell cytotoxic activity is not well understood and may be reduced in a response to *T. gondii* infection (10). In the PEC, frequencies of IFNγ+ and cytotoxic (CD107a+) cNK cells increased 5 days after infection. This increase was independent on the parasite loads. However, the numbers of IFNγ+ and CD107a+ cNK cells only increased after infection with non-replicating *cps 1-1* parasites, but not after infection with replicating RH and ME49 parasites. RH and ME49 parasites also triggered increased frequencies, but not numbers, of IFNγ+ and CD107a+ cNK cells in spleen compared with *cps1-1* parasites. cNK cell function was activated more locally with *cps1-1* strain and is in agreement with previously published studies (53). Whether or not the low numbers of IFNγ+ and CD107a+ cNK cells responding to RH and ME49 infection are sufficient to control infection is not clear. Nevertheless, despite the lower cell frequencies and numbers, cNK cells from RH- and ME49-infected mice appeared to produce higher amounts of IFNγ on a per cell basis than cNK cells from *cps1-1*-infected mice.

The appearance of polyfunctional (IFNγ+CD107a+) cNK cells in a response to *T. gondii* parasites may be advantageous for the resolution of the infection. We observed polyfunctional cNK cells to increase in frequency and numbers in peritoneum 5 days after *cps 1-1* infection. RH and ME49 infection elicited reduced cNK cell polyfunction. Polyfunctional cNK cells responses are advantageous during HIV (28, 29). The fact that the highest numbers of bifunctional cNK cells were found after *cps 1-1* infection and the lowest, after RH infection, could potentially correlate with the outcome of these types of infection. Nevertheless, whether polyfunctional cNK cells are superior to the monofunctional cNK cells during *T. gondii* infection needs to be further investigated.

### cNK Cell Phenotype

Conventional natural killer cells can be classified based on germline-encoded activating and inhibitory receptor expression. These receptors recognize stress-induced or pathogen-specific ligands and MHC I molecules, respectively. The distribution of cNK cell subpopulations, based on activating and inhibitory receptor expression, responding to infection is not affected by the *T. gondii* strain. *T. gondii* infection stimulated increased frequencies of Ly49H+ and Ly49D+ cNK cells, decreased frequencies of CD94+NKG2A+ cNK cells and no change in Ly49I+ cNK cells at the site of infection. The change in frequency of specific cNK cell subsets was not significantly different when compared between three strains. We found that the frequency of Ly49H+ and Ly49D+ cNK cells increased at the infection site. In contrast, the expression level of the activating receptor NKG2D decreased after infection. This is supported by a previous study where peritoneal cNK cells also showed decreased NKG2D expression during acute ME49 infection (54). Interestingly, NKG2D has been shown to be involved in the regulation of DCs by cNK cells during *T. gondii* to promote CD8 T cell responses (55). Whether different *T. gondii* strains affect cNK cell help of DCs and CD8 T cell responses will be important to investigate.

Since similar changes in frequencies of activating receptor expressing cNK cells occurred after infection with all three parasite strains, cNK cell responses may be independent of activating ligands for these receptors. cNK cells can expand in two phases: first *via* cytokines and second *via* recognition of activating ligands in response to infection giving rise to dominant cNK cell populations (32). Therefore, we hypothesize that the cNK cell response to *T. gondii* infection is primarily cytokine-driven rather than ligand-driven, since no specific population appears to increase over others during parasite infection.

Conventional natural killer cells acquire full functional potential *via* a process called licensing (56). Licensed cNK cells express array of inhibitory receptors, including Ly49I and heterodimeric CD94/NKG2A, that recognize classical MHC I and non-classical MHC I (Qa-1), respectively. Unlicensed cNK cells (those not expressing inhibitory receptors) can dominate acute responses to viral infections (57). Our data show that the frequency of Ly49I+ NK cells did not change at the site of infection; however, the frequency of CD94/NKG2A cNK cell subset decreased after the infection. These data suggest that an unlicensed rather than licensed cNK cell population is responding to *T. gondii* infection. These changes occurred regardless of parasite strain. One reason inhibitory receptor expressing cNK cells can change in number is through recognition of MHC Class I (58). Whether *T. gondii* parasites alter classical and non-classical MHC I expression on cells is not clear (59, 60).

Despite cNK cell subset changes after *T. gondii* infection, all the subsets were almost equally distributed in the IFNγ-producing cNK cell population. The changes in numbers of IFNγ-producing cNK cells expressing activating and inhibitory receptors were reminiscent of the changes in numbers of bulk IFNγ-producing cNK cells. Thus, there was no dominant cNK cell subset identified by the phenotypic analysis of IFNγ+ cNK cells. This is different from other infections, including MCMV, HCMV, HIV, Hantavirus, and Chikungunya infections where dominant cNK cell populations are found (17–23). Since we did not find the dominant cNK cell population responding to *T. gondii* infection and all the strains triggered similar changes in distribution of cNK cell subsets at the site of infection, we hypothesize that cNK cell response to *T. gondii* infection is global, cytokine-driven, rather than specific to *T. gondii*-induced cNK cell activating ligands.

### cNK Cell Maturation

Infection with replicating *T. gondii* parasites negatively impacts cNK cell maturation. Peripheral cNK cell maturation is marked by downregulation of CD27 and upregulation of CD11b and KLRG1. At the infection site, we observed an increase in highly mature CD27−CD11b+ cNK cells after infection with ME49 and *cps1-1* parasites. In contrast, after RH infection in both sites and after ME49 infection in spleen, there were fewer mature cNK cells present. This could be explained by the death of mature cells or/and altered cNK cell development. The death of mature cNK cells induced by the infection can contribute to the decrease in the mature cNK cell compartment, but does not explain the increase in numbers of immature cNK cells. Therefore, one possible explanation may be that high level inflammation induced by virulent parasites facilitated the premature escape of cNK cells from the bone marrow, the primary site for cNK cell development. For example, CCL3 has been shown to overcome CXCL12-induced retention of CD11b^low^ cNK cells in the bone marrow and recruit these cells to the blood (61). It will be important to address whether increase in immature cNK cells compartment in peritoneum and spleen after the infection with virulent *T. gondii* parasites is due to the altered cNK cell development in bone marrow. The presence of the incompetent immature CD11b− cNK cells at the expense of mature and functional CD11b+ cNK cells after RH and ME49 infections, and the opposite after *cps1-1* immunization, could be associated with the outcomes of the infections. We hypothesize that the lack in peripheral mature cNK cells contributes to the susceptibility to *T. gondii* infection.

### Cytokine Milieu, Parasite Burden, and cNK Cell Response

Cytokines were shown to initiate proliferation of bulk cNK cells followed by selection of a specific cNK cell population responding to MCMV infection (33). We did not find dominant cNK cell population by receptor phenotype responding to *T. gondii* infection. cNK cell response to *T. gondii* infection seems to be broad, non-specific and is more likely to be driven by cytokines. cNK cell responses are dependent upon IL-12 after *T. gondii* infection (6, 42). As expected, at the site of infection, the levels of proinflammatory cytokines IL-12, IFNγ, and IL-17 were increased, and the level of immunoregulatory cytokine IL-10 was decreased, 5 days after infection with three *T. gondii* strains. In spleen, both IL-12 and IL-10 cytokines were elevated after infection with replicating strains, in contrast to non-replicating *cps1-1* strain that did not induce this change. IL-12 concentration was the highest after the infection with highly virulent RH parasites, followed by moderately virulent ME49 infection, and the lowest IL-12 production was induced by attenuated *cps 1-1* infection. This is supported by the fact that, 5 days after infection, RH parasite numbers were more abundant than ME49 and *cps1-1* parasites. Proinflammatory cytokine overproduction induced by high parasite numbers after infection with virulent *T. gondii* strains leads to higher mortality rate due to the tissue necrosis-, immunopathology- (50–52, 62), and activation-induced cell death (63). The levels of IL-12 and IFNγ could negatively impact the cNK cell population similar to adaptive immune cell populations as previously shown.

IL-1β production increased after *cps1-1* infection in PEC. This correlates well with the quality of cNK cell responses at that site compared with RH and ME49. It has been shown that IL-1β boosts IFNγ production (64–66) and favors cNK cell development from their precursors (67). IL-1β is important for IL-12 induction of IFNγ production in cNK cells during *T. gondii* infection (68). Whether IL-1β can be impacted by parasite virulence needs to be further investigated.

Infection with all parasite strains stimulated increased levels of IL-17 at the site of infection. IL-17 is essential for the control of infection (69), and cNK cells are the relevant source of IL-17 during acute toxoplasmosis (70). The decrease in frequency and numbers of cNK cells in spleen after infection with replicating parasites may also be affected by IL-10 production, since IL-10 is an immunoregulatory cytokine that balances the proinflammatory immune responses that can be detrimental to the host (52, 71). In peritoneum, where we observed the most robust cNK cell response, IL-10 was decreased after infection with all the strains. IL-10 was increased in spleen after infection with virulent RH and ME49 parasites compared with *cps1-1* parasites.

## Conclusion

In the present study, we, in detail, characterized cNK cell response to *T. gondii* infection. Infection with Type I RH parasites and Type II ME49 parasites triggers high level of inflammation associated with robust production of IL-12 in PEC, spleen and blood. This may promote reduction in splenic cNK cell compartment. This decrease in number may be due to activation induced cell death and/or migration of cells to the infection site. All three *T. gondii* strains mediated increase in mono- and polyfunctional cNK cells at the infection site. Infection with different parasite strains stimulated similar changes in cNK cell subsets expressing activating and inhibitory receptors. Whether the changes in cNK cell phenotype are associated with the cytokine signaling received and/or the change in the ligand expression is not clear. We found that attenuated *T. gondii* infection could stimulate an increase in cNK cell maturation. However, infection with replicating parasites reduced mature cNK cells and increased immature cNK cell output. Thus, many open questions remain about how *T. gondii* parasites may impact cNK development and promote early control of infection. Understanding how infection with *T. gondii* parasites of different virulence impacts cNK cell response is important for the development of the therapies to prevent and treat *T. gondii* infection in humans.

## Author Contributions

DI designed the study, performed the experiments, analyzed the data, and drafted and revised the manuscript. RF performed the experiments and analyzed the data. JG designed the study and drafted and revised the manuscript. All authors read and approved the final manuscript.

## Conflict of Interest Statement

The authors declare that the research was conducted in the absence of any commercial or financial relationships that could be construed as a potential conflict of interest.
